# Patrinoside and Patrinoside A from *Patrinia scabiosaefoli*a Improve Insulin Resistance by Inhibiting NF-*κ*B, MAPK Pathways and Oxidative Stress in RAW264.7 and 3 T3-L1 Cells

**DOI:** 10.1155/2023/9069645

**Published:** 2023-01-24

**Authors:** Zhenhua Liu, Mengke Wang, Yuhang Liu, Mengjie Ren, Xuefeng Xi, Shiming Li, Wenyi Kang

**Affiliations:** ^1^National R & D Center for Edible Fungus Processing Technology, Henan University, Kaifeng 475004, China; ^2^Functional Food Engineering Technology Research Center, Henan, Kaifeng 475004, China; ^3^Joint International Research Laboratory of Food & Medicine Resource Function, Henan Province, Kaifeng 475004, China; ^4^College of Physical Education, Henan University, Henan, Kaifeng 475004, China; ^5^Shenzhen Research Institute of Henan University, Shenzhen 518000, China

## Abstract

*Patrinia scabiosaefolia*, as traditional food and medicine plant, was used to treat appendicitis, enteritis, and hepatitis for thousand years in China. Patrinoside and patrinoside A isolated from *P. scabiosaefolia* could significantly improve insulin resistance (IR) by activating PI-3 K/AKT signaling pathway in our previous study. Since IR is closely related to inflammation, their anti-inflammatory activities in RAW264.7 inflammatory model induced by LPS and in 3 T3-L1 IR inflammatory model induced by TNF-*α* were evaluated to identify whether the effects on improving IR related to anti-inflammatory activity. In RAW264.7 cells, patrinoside and patrinoside A significantly inhibited the transcription and secretion of inflammatory mediators NO, TNF-*α*, and IL-6. Western blot analysis showed that the significant inhibition of phosphorylation of I*κ*B and P65 and P38, ERK and JNK suggested that the effects were exerted through NF-*κ*B pathway and MAPK pathway. In 3 T3-L1 cells, patrinoside and patrinoside A also inhibited the activation of NF-*κ*B and MAPK pathways through inhibiting the transcriptions of inflammatory cytokines IL-6 and chemokines MCP-1 and MIP-1*α*. These events resulted in the inhibition of macrophages migration to adipocytes. In addition, patrinoside and patrinoside A ameliorated oxidative stress by inhibiting ROS release in LPS-stimulated RAW264.7 cells. In conclusion, patrinoside and patrinoside A could active PI-3 K/AKT pathway, inhibit NF-*κ*B pathway, MAPK pathway, and improve oxidative stress, which showed multipathways on improving IR. These results provided the scientific basis for material basis and mechanism on improving IR of *P. scabiosaefolia*.

## 1. Introduction

Insulin resistance (IR) refers to the decreased sensitivity of peripheral tissues (muscle, liver, and adipose tissues) to insulin, mainly manifested as the impairment of glucose uptake and utilization [1]. The pathogenesis of IR is very complex, and a large number of basic studies and clinical trials have confirmed that IR is related to inflammation, oxidative stress, and abnormal intracellular signaling pathways [2].

Since the correlation between inflammatory and IR was first proposed in the early 1990s, a large number of studies were conducted and confirmed [3]. There is a complex cross relationship between inflammatory signal transduction pathway and insulin signal pathway. Inflammatory factors affect the normal conduction of insulin, and eventually occur or aggravate IR [4]. The mechanism of IR induced by inflammatory involves multiple signaling pathways, such as IKK/NF-*κ*B pathway, JNK pathway, SOCS pathway, and PKC pathway. Inflammatory factors activated JNK and IKK, which resulted in the phosphorylation of IRS-1 serine, inhibiting the normal tyrosine phosphorylation of IRS-1. Thus, these events ultimately interfered GLUT4 membrane migration and inhibited glucose transport [5, 6]. At the same time, MAPK and IKK/NF-*κ*B pathways activated by inflammatory factors could promote the entry of NF-*κ*B and AP-1 into the nucleus, and regulated and promoted the expression of proinflammatory factors and chemokines, ultimately aggravating IR [7]. In addition, adipose tissue in IR state could induce infiltration of macrophages and promote the release of inflammatory factors to aggravate IR, forming a vicious cycle [8].

In recent years, many clinical experiments have proved that anti-inflammatory is one of the effective strategies to treat IR which is also a hotspot of current research. IL-1 receptor antagonist IL-1RA, which has been approved by the US Food and Drug Administration (FDA) for the treatment of T2DM, is a natural anti-inflammatory factor. Multiple studies have demonstrated that IL-1RA could reduce blood glucose levels by promoting insulin secretion by islet *β* cells and improving insulin sensitivity of adipose tissue [9–11]. Also, many plant-derived iridoids exerted hypoglycemic activity and anti-inflammatory effects at the same time. Catalpol, the main active component from *Rehmannia glutinos*, can not only exert anti-inflammatory effects in LPS-induced bovine endometrial epithelial cells (bEECs) and mouse endometritis by inhibiting the activation of TLR4/NF-*κ*B pathway but also through inhibition of the RAGE/RhoA/ROCK pathway reduces inflammation in diabetic nephropathy [12, 13]. It was reported that iridoid glycoside from *Corni Fructus* reduced inflammation and oxidative stress by inhibiting NF-*κ*B and enhancing PI3K/AKT signaling pathway, and significantly alleviated hyperglycemia and insulin resistance in T2DM with NAFLD mice [14].

**Figure 1:**
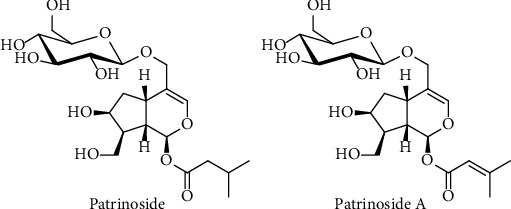
Structures of patrinoside and patrinoside A from *P. scabiosaefolia.*

*Patrinia scabiosaefolia* Fisch. ex Trev. belonging to Valerianaceae family, is widely distributed throughout the country in China [15]. It was the first record in “*Shen Nong's Herbal Classic*”, used to treat appendicitis, enteritis, hepatitis, etc. Since *P. scabiosaefolia* has strong putrid odor, it is named as “Baijiang Cao” in Chinese name, whose main chemical constituents is iridoids [16]. In our previous studies, two iridoids patrinoside and patrinoside A (Figure 1) isolated from *Patrinia scabiosaefolia*, which could improve insulin resistance (IR) of adipocytes by activating PI3K/AKT signaling pathway [17]. And *Patrinia scabiosaefolia*, as a traditional food and medicine plant, was reported to have anti-inflammatory activity [18]. Therefore, in order to further explore whether the mechanisms on improving IR of patrinoside and patrinoside A were associated with anti-inflammatory activity, their anti-inflammatory activities and mechanisms were evaluated in LPS-induced RAW264.7 and TNF-*α*-induced 3 T3-L1 cells.

## 2. Materials and Methods

### 2.1. Materials

Patrinoside and patrinoside A were separated and purified from *P. scabiosaefolia* by National R & D Center for Edible Fungus Processing Technology, Henan University.

### 2.2. Cell Culture

The culture method of RAW264.7 cells was the same as reported by Gao et al. [19]. The method of culturing 3 T3-L1 cells and inducing differentiation into mature adipocytes is consistent with previous reports [18].

### 2.3. Cell Viability Assay

Consistent with previous reports [18–20], MTT assay was used to detect the effects of patrinoside and patrinoside A on RAW264.7 and 3 T3-L1 cell viability, respectively.

### 2.4. Determination of Cytokines

RAW264.7 cells were pretreated with patrinoside and patrinoside A for 1 h, then LPS was added for 24 h, and the supernatant was collected. The contents of NO, IL-6 and TNF-*α* were detected according to the kit instructions.

Mature adipocytes were induced with TNF-*α* for 24 h, and then treated with patrinoside and patrinoside A for 24 h. Supernatant was collected, and IL-6 and MCP-1 contents were detected according to the kit instructions.

### 2.5. Determination of ROS Level in RAW264.7cells

RAW264.7 cells were divided into groups and treated in the same way as the above method. The treated cells were added with serum-free DMEM containing DCFH-DA and cultured at 37°C for 25 min. The probe was removed by washing lightly with PBS for 3 times. ROS levels were detected by flow cytometry after passing 300 mesh nylon screens.

### 2.6. Transwell Analysis

After RAW264.7 cells were treated according to the above methods, cells from blank group, model group, and drug-treated group were collected and seeded onto the Transwell inserts (2 × 10^5^ cells/well). The supernatant of mature adipocytes incubated for 24 h was transferred to 24-well plates containing inserts. And then it was incubated in an incubator for 24 h.

After 3 T3-L1 cells were treated according to the above method, supernatants of blank group, model group, and drug-treated group were placed in 24-well plates, normal RAW264.7 cells were inoculated in the transwell inserts (2 × 10^5^ cells/well), and then incubated for 24 h.

Transwell inserts were fixed with 4% formaldehyde for 25 min, and then washed with PBS; the internal cells were erased, and stained with crystal violet for 20 min. The cells were cleaned with PBS again, and photos were taken with inverted microscopes of 20×.

### 2.7. Western Blot and qRT-PCR Analysis

RAW264.7 and 3 T3-L1 cells were treated according to the above method and collected. Western Blot and qRT-PCR analysis were consistent with previous reports [18, 19].

### 2.8. Statistical Analysis

All data were presented as mean ± standard deviation (SD). Results were analyzed by SPSS19.0 for one-way ANOVA. *P* < 0.05 was considered statistically significant.

## 3. Result

### 3.1. Effects of Patrinoside and Patrinoside A on LPS-Induced RAW2647 Cells

#### 3.1.1. Effects of Patrinoside and Patrinoside A on Viability of RAW264.7 Cells

**Figure 2:**
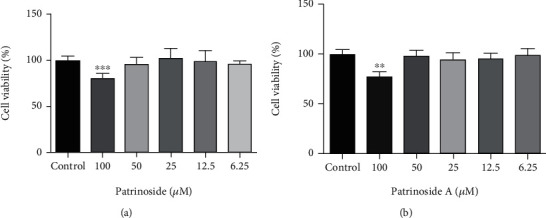
The effects of patrinoside (a) and patrinoside A (b) on the viability of RAW264.7 cells. ^∗∗∗^*P* < 0.001, ^∗∗^*P* < 0.01 versus control group.

MTT assay was used to evaluate the RAW264.7 cytotoxicity of patrinoside and patrinoside A. In Figure 2, patrinoside and patrinoside A did not showed significant effects on the viability of RAW264.7 cells when the concentration was less than 50 *μ*M. Therefore, 12.5, 25, and 50 *μ*M were selected as low, medium, and high doses of patrinoside and patrinoside A, respectively.

#### 3.1.2. Effects of Patrinoside and Patrinoside A on NO Secretion and iNOS Expression in RAW264.7 Cells

**Figure 3:**
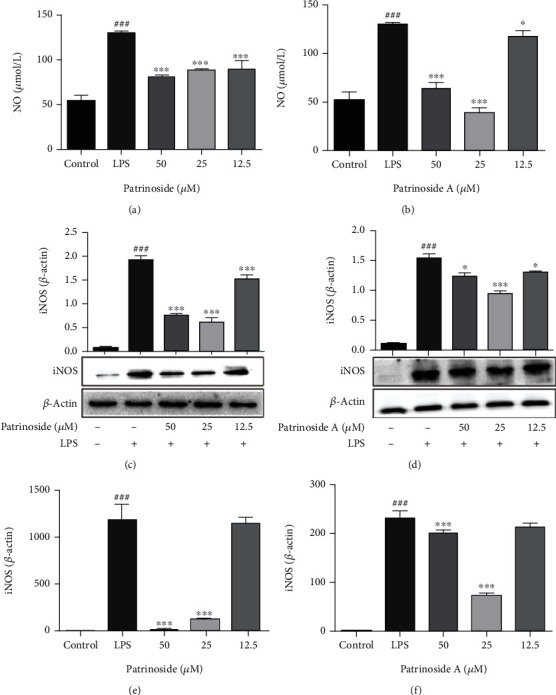
The effects of patrinoside (a, c, e) and patrinoside A (b, d, f) on NO production, iNOS protein expression, and mRNA transcription in LPS-induced RAW264.7 cells. (a, b) NO production; (c, d) iNOS protein expression; (e, f) iNOS mRNA expression. ^###^*P* < 0.001 versus control group, ^∗∗∗^*P* < 0.001, ^∗^*P* < 0.05 versus LPS group.

NO is indispensable as a proinflammatory mediator in the development of inflammation. The effects of patrinoside and patrinoside A on NO secretion in LPS-induced RAW264.7 cells were determined by nitrate reductase. In Figures 3(a)–3(b), the content of NO in normal cells was low, but after LPS stimulation, the content of NO increased significantly (*P <*0.001), indicating that the inflammatory model was successfully established. Compared with LPS group, both patrinoside and patrinoside A significantly inhibited NO secretion at 25 and 50 *μ*M (*P* < 0.001), and when the concentration lowed to 12.5 *μ*M, the effects of patrinoside (*P <0.001*) was better than patrinoside A (*P* < 0.05), and the effects of patrinoside was showed in a dose-dependent manner. The results indicated that patrinoside and patrinoside A had significant anti-inflammatory activity.

NO secretion is regulated by iNOS, so the effects of patrinoside and patrinoside A on iNOS protein expression and mRNA transcription were further determined by Westen Blot and qRT-PCR. Compared with control group, iNOS protein level could significantly increase after LPS stimulation, while patrinoside and patrinoside A could significantly inhibit its expression (*P* < 0.001) (Figures 3(c)– 3(d). Similarly, patrinoside and patrinoside A significantly downregulated the transcription level of iNOS mRNA at 50 and 25 *μ*M (*P* < 0.001), and patrinoside showed a more obvious inhibitory effect (Figures 3(e)–(f) compared with LPS group. Thus, patrinoside and patrinoside A could inhibit the mRNA transcription of iNOS and downregulate its protein expression, and then reduce the LPS induced NO release in RAW 264.7 cells, thereby improving the inflammatory response of macrophages.

#### 3.1.3. Effects of Patrinoside and Patrinoside A on Secretion and Transcription of Inflammatory Factors in RAW264.7 Cells

**Figure 4:**
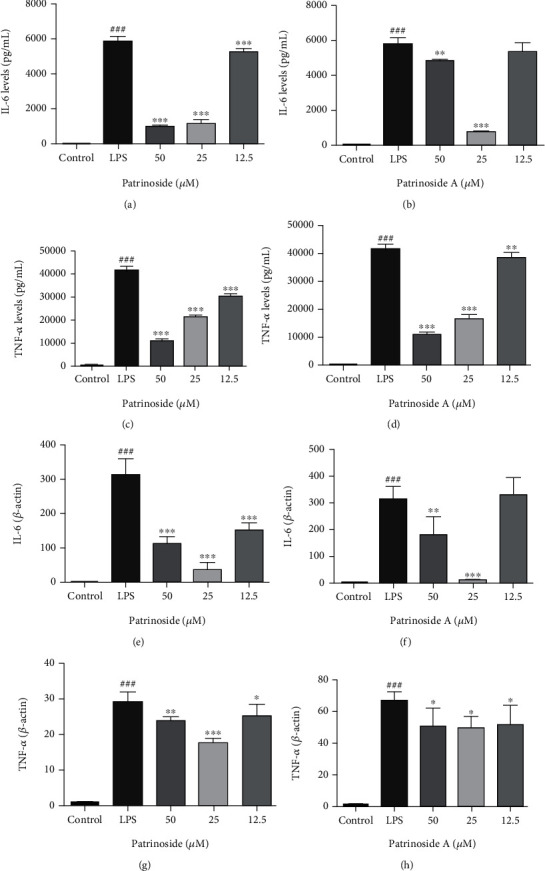
The effects of patrinoside (a, c, e, g) and patrinoside A (b, d, f, h) on IL-6 and TNF-*α* production and mRNAexpression in LPS-induced RAW264.7 cells. (a, b) IL-6 production; (c, d) TNF-*α* production; (e, f) IL-6 mRNA expression; (g, h) TNF-*α* mRNA expression. ^###^*P* < 0.001 versus control group, ^∗∗∗^*P* < 0.001, ^∗∗^*P* < 0.01, ^∗^*P* < 0.05 versus LPS group.

The extent of the inflammatory response is partly reflected by two important inflammatory factors, TNF-*α* and IL-6. ELISA was used to detect the effects of patrinoside and patrinoside A on the secretion of proinflammatory factors in LPS-stimulated RAW264.7 cells in Figures 4(a)–4(d). Compared with the control group, TNF-*α* and IL-6 levels increased sharply after LPS stimulation (*P* < 0.001), but decreased significantly in the drug-treated group. For IL-6, patrinoside at different concentrations (25, 50, and 100 *μ*M) showed a very significant downregulation trend (*P* < 0.001) in a dose-dependent manner. Patrinoside A did not significantly inhibit LPS-induced IL-6 at 12.5 *μ*M (*P* > 0.05), but showed extremely significant inhibition at 25 and 50 *μ*M, and the inhibitory effect was the best at 25 *μ*M (*P* < 0.001). In addition, both patrinoside and patrinoside A significantly inhibited LPS-induced TNF-*α* secretion (*P* < 0.001) in a dose-dependent manner.

On this basis, the effects of patrinoside and patrinoside A on LPS-induced proinflammatory factor mRNA transcription were detected by qRT-PCR. In Figures 4(e)–4(h), patrinoside and patrinoside A inhibited TNF-*α* and IL-6 mRNA levels to varying degrees (*P* < 0.001, *P* < 0.01, *P* < 0.05). In general, patrinoside had a better downregulation effect on TNF-*α* and IL-6 mRNA than that of patrinoside A, and its inhibition effect was the best in the medium dose group at 25 *μ*M. The results indicated that patrinoside and patrinoside A improved the inflammatory response of macrophages by inhibiting the transcription of TNF-*α* and IL-6 mRNA, thereby avoiding LPS-induced secretion of large amounts of proinflammatory factors.

#### 3.1.4. Effects of Patrinoside and Patrinoside A on the Expression of Related Proteins of NF-*κ*B and MAPK Pathways in RAW264.7 Cells

**Figure 5:**
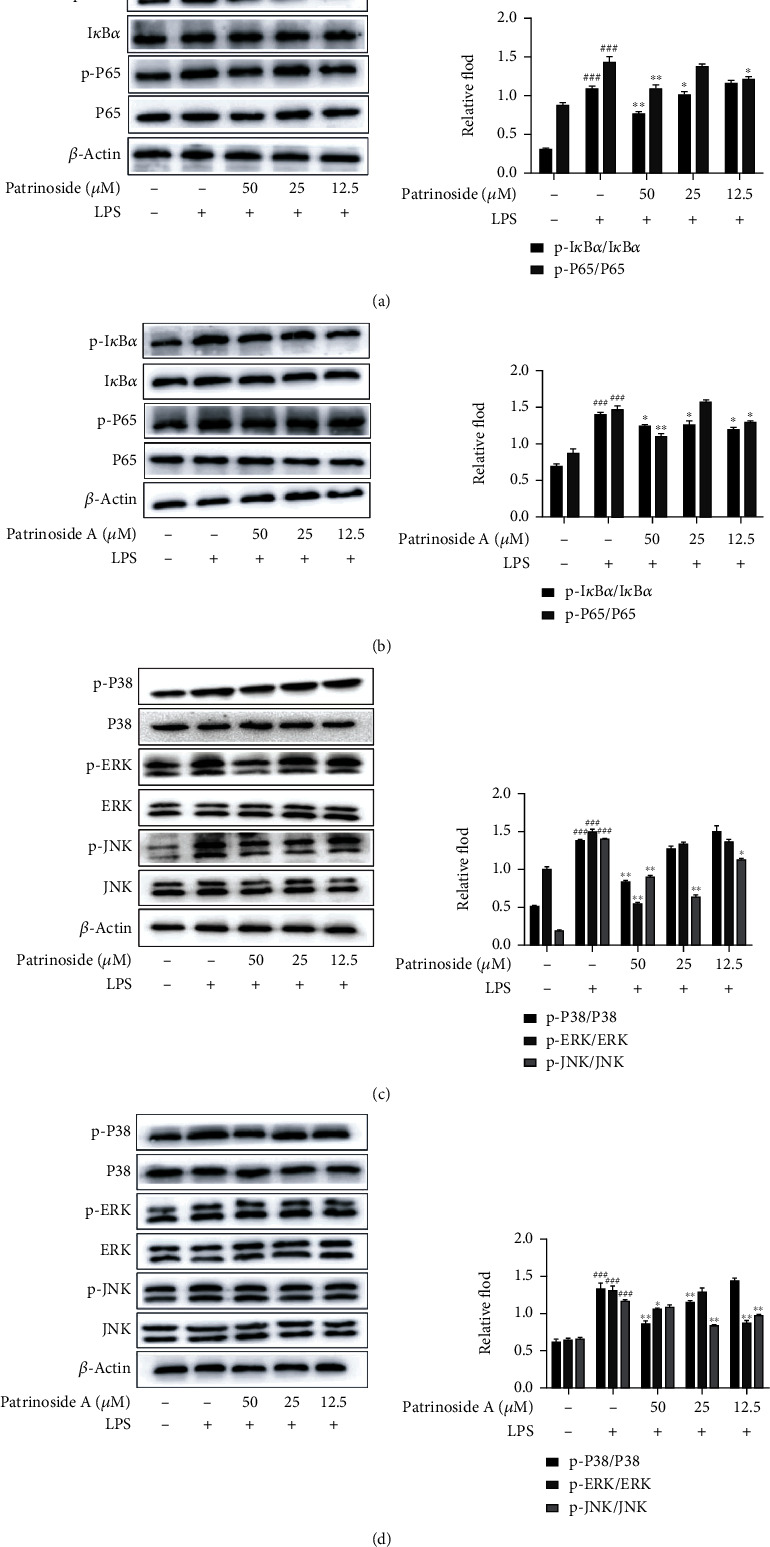
The effects of patrinoside and patrinoside A on NF-*κ*B (a, b) and MAPK (c, d) signal pathways in LPS-induced RAW264.7 cells. ^###^*P* < 0.001 versus control group, ^∗∗^*P* < 0.01, ^∗^*P* < 0.05 versus LPS group.

To further clarify the anti-inflammatory mechanism of patrinoside and patrinoside A, Western Blot was used to detect the effects of patrinoside on NF-*κ*B and MAPK signaling pathways. In Figures 5(a) and 5(b), the NF-*κ*B pathway was activated and phosphorylation of I*κ*B and P65 was significantly increased after LPS stimulation compared with the control group (*P* < 0.001). Patrinoside significantly reduced the phosphorylation ratio of I*κ*B at 50 and 25 *μ*M (*P* < 0.01, *P <*0.05), and inhibited the phosphorylation of P65 at 50 and 12.5 *μ*M (*P <*0.01, *P* < 0.05). Patrinoside A inhibited p-I*κ*B expression in three dose groups (*P* < 0.05) and p-P65 expression at 50 and 12.5 *μ*M.

Meanwhile, in Figures 5(c) and 5(d), patrinoside and patrinoside A inhibited the activation of LPS-stimulated MAPK pathway compared with LPS group. Patrinoside significantly inhibited phosphorylation of P38, ERK, and JNK at 50 *μ*M (*P* < 0.01), and also downregulated JNK phosphorylation at 25 (*P* < 0.01) and 12.5 (*P* < 0.05) *μ*M. Patrinoside A significantly inhibited p-P38 expression at 50 and 25 *μ*M (*P* < 0.01), inhibited p-ERK expression at 25 (*P* < 0.05), and 12.5 (*P* < 0.01) *μ*M, and downregulated p-JNK expression at 25 and 12.5 *μ* (*P* < 0.01). The results suggested that patrinoside and patrinoside A inhibit the inflammatory response of RAW264.7 cells by inhibiting the activation of NF-*κ*B and MAPK signaling pathways.

#### 3.1.5. Effects of Patrinoside and Patrinoside a on ROS in RAW264.7 Cells

**Figure 6:**
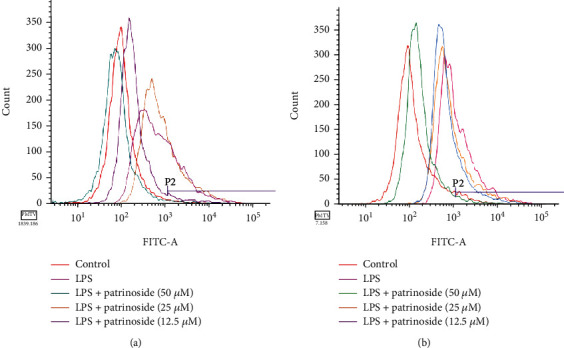
The effects of patrinoside (a) and patrinoside A (b) on ROS induced by LPS.

LPS can cause macrophages to produce a large amount of ROS, resulting in oxidative stress damage. In Figure 6, compared with the control group, the wave peak shifted significantly to the right after LPS stimulation, and the average fluorescence intensity increased, indicating a sharp increase in intracellular ROS, which proved that LPS-induced RAW264.7 cells underwent oxidative stress. Patrinoside at 50 *μ*M and 12.5 *μ*M significantly shifted the wave peak to the left and decreased the average fluorescence intensity, while it showed no significant difference from LPS group at 25 *μ*M. For patrinoside A, compared with LPS group, the three concentrations showed a left-shift trend, which could inhibit production of ROS. For patrinoside and patrinoside A, it showed the best effects on inhibiting ROS production when the concentration was 50 *μ*M. Thus, both patrinoside and patrinoside A could reduce the LPS-induced intracellular ROS level.

### 3.2. Effects of Patrinoside and Patrinoside A on TNF-*α*-Induced Inflammation of 3 T3-L1 Cells

#### 3.2.1. Effects of Patrinoside and Patrinoside A on Viability of 3 T3-L1 Cells

**Figure 7:**
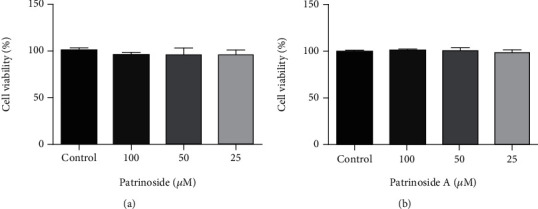
The effects of patrinoside (a) and patrinoside A (b) on the viability of 3 T3-L1 cells.

MTT assay was used to detect the effects of patrinoside and patrinoside A on 3 T3-L1 preadipocyte toxicity. In Figure 7, patrinoside and patrinoside A showed no cytotoxicity at the concentration of 25~100 *μ*M.

#### 3.2.2. Effects of Patrinoside and Patrinoside A on Secretion and Transcription of Inflammatory Factors in Adipocytes

**Figure 8:**
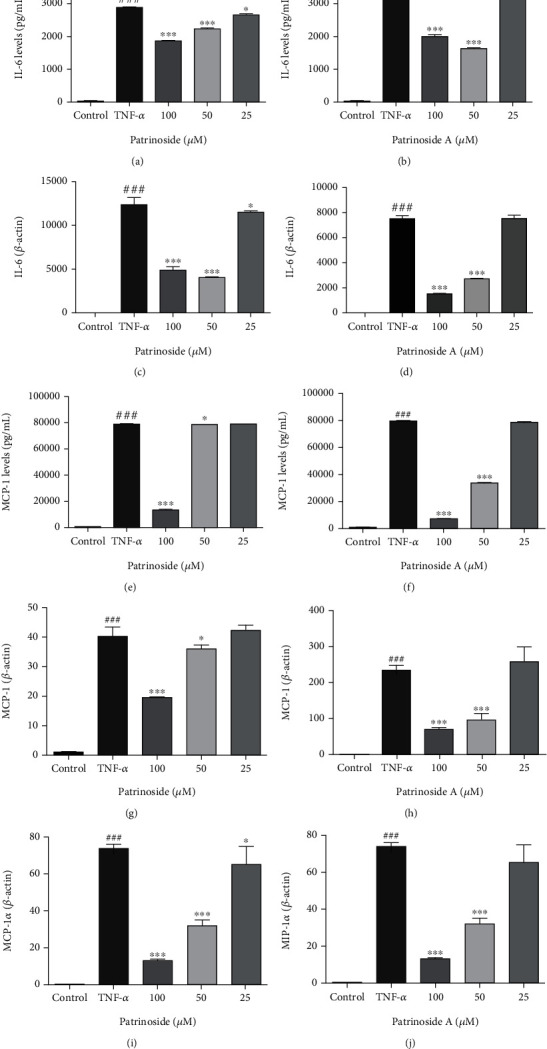
The effects of patrinoside (a, c, e, g, i) and patrinoside A (b, d, f, h) on cytokines production and mRNA expression in TNF-*α* -induced adipocytes. (a, b) IL-6 production; (c, d) IL-6 mRNA expression; (e, f) MCP-1 production; (g, h) MCP-1 mRNA expression; (i, j) MIP-1*α* mRNA expression. ^###^*P* < 0.001 versus control group, ^∗∗∗^*P* < 0.001, ^∗^*P* < 0.05 versus TNF-*α* group.

Compared with the control group, the secretion and transcription level of IL-6 in the LPS group were significantly increased (*P* < 0.001), which indicated that the adipocyte inflammation model was established. After treatment with patrinoside and patrinoside A, IL-6 secretion could significantly decrease at 100 and 50 *μ*M (*P* < 0.001), and when the concentration lowed to 25 *μ*M, patrinoside was still valid (*P* < 0.05). Similarly, patrinoside and patrinoside A inhibited IL-6 mRNA transcription to varying degrees, reaching extremely significant levels at 100 and 50 *μ*M (*P* < 0.001) in Figures 8(a)–8(h). Both patrinoside and patrinoside A inhibited the secretion and transcription of MCP-1 at 100 and 50 *μ*M (*P* < 0.001, *P* < 0.05), and patrinoside A showed better effect. In addition, both patrinoside and patrinoside A at 100 and 50 *μ*M significantly inhibited MIP-1*α* transcription (*P* < 0.001), and patrinoside also showed significant inhibition at 25 *μ*M (*P* < 0.05) in Figures 8(i) and 8(j). The results suggested that patrinoside and patrinoside A can improve TNF-*α* induced adipocyte inflammation by inhibiting the secretion and mRNA transcription of inflammatory factors and chemokines.

#### 3.2.3. Effects of Patrinoside and Patrinoside A on NF-*κ*B and MAPK Signaling Pathways Induced by TNF-*α* in Adipocytes

**Figure 9:**
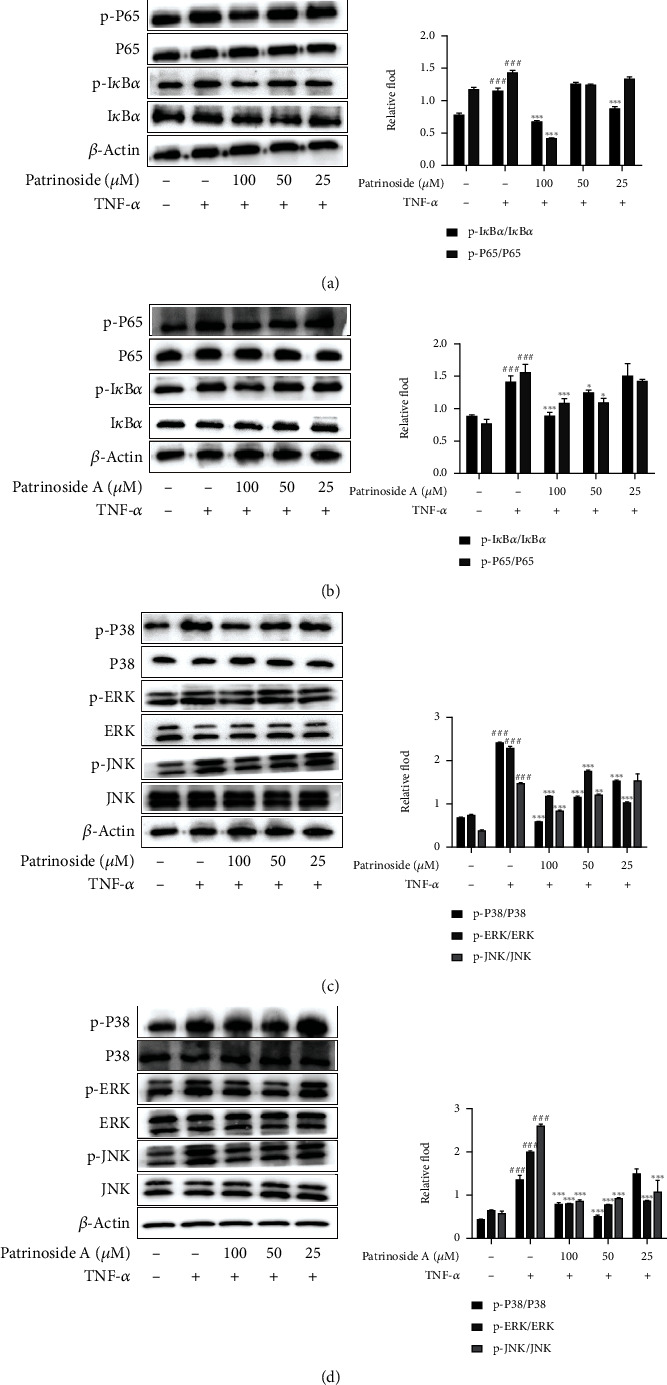
The effects of patrinoside and patrinoside A on NF-*κ*B (a, b) and MAPK (c, d) signal pathways in TNF-*α* -induced adipocytes. ^###^*P* < 0.001 versus control group, ^∗∗∗^*P* < 0.001^∗∗^*P* < 0.01,  ^∗^*P* < 0.05 versus TNF-*α* group.

To explore the mechanism of patrinoside and patrinoside A on improving inflammation of adipocytes, Western Blot was used to detect key proteins of NF-*κ*B and MAPK (Figure 9). After TNF-*α* stimulation, NF-*κ*B pathway was activated. Treatment with patrinoside, it was significantly inhibited p-I*κ*B expression at 100 *μ*M (*P* < 0.001) and phosphorylation of P65 at 100 and 25 *μ*M (*P* < 0.001). Also, patrinoside A downregulated the expression of p-P65 and p-I*κ*B at 100 (*P* < 0.001) and 50 *μ*M (*P* < 0.05). Patrinoside and patrinoside A also inhibited TNF-*α*-induced phosphorylation of P38, ERK, and JNK proteins. Patrinoside significantly inhibited p-P38 and P-ERK expression at all concentrations (*P* < 0.001), and p-JNK expression was significantly inhibited at 100 (*P* < 0.001) and 50 *μ*M (*P* < 0.01). Patrinoside A significantly inhibited the phosphorylation of P38, ERK, and JNK at 100 and 50 *μ*M (*P* < 0.001), and also inhibited the expression of P-ERK and P-JNK at 25 *μ*M (*P* < 0.001). Therefore, patrinoside and patrinoside A may ameliorate IR inflammation in adipocytes by inhibiting the activation of NF-*κ*B and MAPK signaling pathways.

#### 3.2.4. Effects of Patrinoside and Patrinoside A on Macrophage Infiltration of Adipocytes

**Figure 10:**
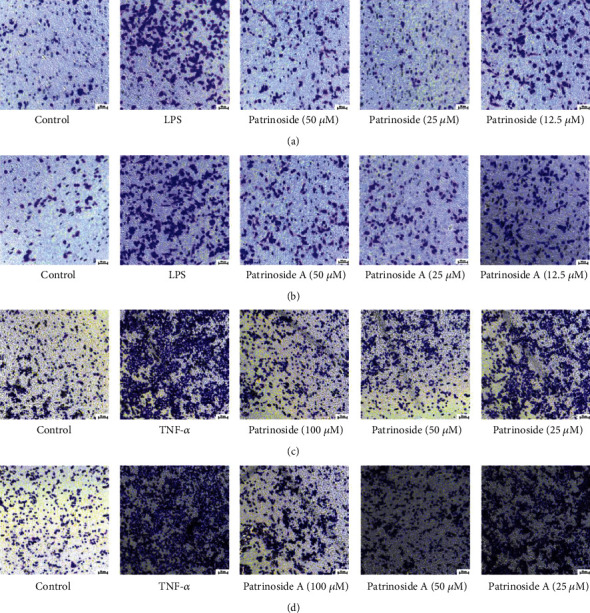
Effects of patrinoside and patrinoside A on migration of RAW264.7 cells to 3 T3-L1 adipocytes (20×). (a, b) migration of LPS-induced macrophages to adipocytes; (c, d) Migration of macrophages to TNF-*α*-induced adipocytes.

Excessive secretion of inflammatory factors and chemokines can induce macrophages to infiltrate adipose tissue. Therefore, transwell was used to observe the effects of patrinoside and patrinoside A on the migration of macrophages to adipocytes. LPS stimulated RAW264.7 cells to produce an inflammatory response, resulting in the promotion the migration from inflammatory macrophages to adipocytes (Figures 10(a) and 10(b)). However, after the treatment of patrinoside and patrinoside A at high and medium concentrations, the number of macrophages to adipocytes was significantly reduced. The results showed that LPS-induced macrophage migration to adipocytes was inhibited. At the same time, patrinoside and patrinoside A inhibited macrophage infiltration into TNF-*α*-induced adipocytes in Figure 10(c) and 10(d). Combined with the detection results of the above inflammatory factors, patrinoside and patrinoside A may inhibit the transcription levels of cytokines IL-6, MCP-1, and MIP-1*α* and then inhibit their secretion to inhibit the infiltration of RAW264.7 cells into adipocytes and improve the inflammatory response of adipocytes.

## 4. Discussions

Since 1993, when inflammation was proposed to be related to IR, a large number of studies have proved that inflammation is an important factor causing the occurrence or aggravation of IR, and anti-inflammatory treatment of IR has become hotspot in clinical research and new drug development [21]. High doses of salicylates in the last century have been shown to reduce glucose levels in diabetic patients, and to date clinical trials have proved that inflammatory cytokine antagonists such as Infliximab, Etanercept, Anakinra, and Canakinumab can improve insulin sensitivity by inhibiting inflammation [22–25]. In addition, lipid-lowering drug atorvastatin [26] and hypoglycemic drug rosiglitazone [27] have also been found to improve IR status by inhibiting the inflammatory pathway NF-*κ*B.

*P. scabiosaefolia*, as a traditional food and medicine plant, was reported to have anti-inflammatory activity and has the potential to become a medicinal food [28]. The methanol extract of *P. scabiosaefolia* can inhibit the secretion and mRNA expression of proinflammatory factors TNF-*α*, IL-1*β*, and IL-6 [29]. The ethyl acetate fraction of *P. scabiosaefolia* can reduce the contents of NO and IL-6 in RAW264.7 cells induced by LPS, and inhibits the expression of iNOS and COX-2 [30]. Oral administration of *P. scabiosaefolia* can reduce the serum amylase and lipase levels in rats with cholecystokinin (CCK) octapeptide induced acute pancreatitis (AP), and inhibit the expression of proinflammatory cytokines to produce anti-inflammatory effects [31].

Iridoids, as the main chemical constituents of *P. scabiosaefolia*, exhibit various biological activities, such as anti-inflammatory, antioxidant, and antitumor [32–38]. Furthermore, an increasing number of studies have shown that iridoids can improve T2DM through multiple ways [39]. Catalpa, a bioactive ingredient in *Zantedeschia rehmannii* root, can not only improve liver insulin resistance by acting on AMPK/NOX4/PI-3 K/AKT pathways [7] but also improve HFD-induced insulin resistance in mice by reducing adipose tissue inflammation and inhibiting JNK and NF-*κ*B pathways [40]. It was reported that iridoid glycoside from *Corni Fructus* reduced inflammation and oxidative stress by inhibiting NF-*κ*B and enhancing PI3K/AKT signaling pathway, and significantly alleviated hyperglycemia and insulin resistance in T2DM with NAFLD mice [14]. Mo et al. [41] discovered that loganin could inhibit nuclear migration and accumulation of FOXO1 by activating the PI-3 K/AKT pathway, thus protecting the insulin secretion function of INS-1 cells in islets. In addition, it has been found to improve painful diabetic neuropathy by governing oxidative stress, inflammation and insulin sensitivity in diabetic rats [42]. Therefore, based on the hypoglycemic activity of two iridoids (patrinoside and patrinoside A) found in previous studies [17], this experiment established RAW264.7 inflammation model to preliminarily screen the anti-inflammatory activity of patrinoside and patrinoside A. And then TNF-*α* induced 3 T3-L1 cell create insulin resistance inflammatory model to further determine the mechanism on improving IR of patrinode and patrinoside A related to anti- inflammation.

Inflammatory cytokines and inflammatory mediators are massively secreted by macrophages stimulated by LPS, and the development of inflammation can be indirectly reflected by the release of these inflammatory cytokines [43]. In our study, patrinoside and patrinoside A could improve the inflammatory state by inhibiting the secretion of NO, TNF-*α*, and IL-6 and the transcription levels of iNOS, TNF-*α*, and IL-6 mRNA in RAW264.7 cells. At the same time, janus kinase signal transduction and transcription activator (JAK-STAT), mitogen activated protein kinase (MAPK), and nuclear factor kappa B (NF-*κ*B) are important intracellular signaling pathways involved in the inflammatory response. MAPK can convert extracellular signals such as stress and growth factors into activation of intracellular signaling pathways and is considered a potential target for anti-inflammatory therapies due to its involvement in regulating the synthesis of inflammatory mediators at the transcriptional and translational levels [44]. The canonical NF-*κ*B pathway is a key mediator of the inflammatory response, and when abnormally activated, it can regulate the expression of several proinflammatory factors through rapid but transient transcriptional activity [45]. In this study, patrinoside and patrinoside A significantly inhibited the phosphorylation of P65, I*κ*B, ERK, JNK, and P38 proteins in macrophages, further suggesting that patrinoside and patrinoside A could inhibit the transcription and release of inflammatory factors by inhibiting the activation of NF-*κ*B and MAPK pathways, thereby improving the inflammatory state of macrophages.

A large number of ROS are released due to oxidative stress induced by LPS from macrophages and oxidative stress is proved that closely associated with inflammation [46]. ROS can activate the NF-*κ*B pathway and promote the release of inflammatory factors, which in turn can promote the production of ROS, thereby aggravating the oxidative stress damage of cells [47]. In addition, the persistent elevation of ROS caused by inflammation affects ROS and redox signaling, thereby impairing islet *β*-cell function [48]. In this study, patrinoside and patrinoside A also downregulated the increase of ROS induced by LPS, suggesting that they also had significant antioxidant effects. Therefore, it was speculated that patrinoside and patrinoside A might inhibit the inflammatory response of RAW264.7 cells by inhibiting NF-*κ*B and MAPK pathways and reducing the release of inflammatory factors and ROS to improve IR.

When IR occurs in adipose tissue, a variety of inflammatory factors will be secreted to induce macrophages to infiltrate and release inflammatory factors, thus aggravating the inflammatory response of adipose tissue and further aggravating IR, forming a vicious cycle [49]. Therefore, 3 T3-L1 cells were induced by TNF-*α* to construct IR inflammatory model, to further study the mechanism of patrinoside and patrinoside A in improving adipocyte IR through anti-inflammatory action. The results showed that patrinoside and patrinoside A could inhibit the secretion and transcription of inflammatory factors (IL-6) and chemokines (MCP-1 and MIP-1*α*) of adipose cells through the inhibition of NF-*κ*B and MAPK pathways.

Meanwhile, the inflammatory state of adipose tissue is closely related to the infiltration of macrophages and the secretion of inflammatory factors [8]. Infiltration of macrophages plays an active role in the development of inflammation in adipose tissue, while their overactivation leads to the production of proinflammatory factors that can damage the body [50, 51]. And transwell experiment showed that they could inhibit the migration of macrophages to adipose cells. Therefore, we speculate that patrinoside and patrinoside A inhibit macrophage infiltration into adipose tissue and thus reduce adipose tissue inflammation, which may be related to inhibiting the secretion of inflammatory cytokines and chemokines. In addition, Patrinoside and patrinoside A significantly inhibited the activation of NF-*κ*B and MAPK, which are important pathways of inflammatory IR signal transduction disorders, thereby increasing insulin transduction.

**Figure 11:**
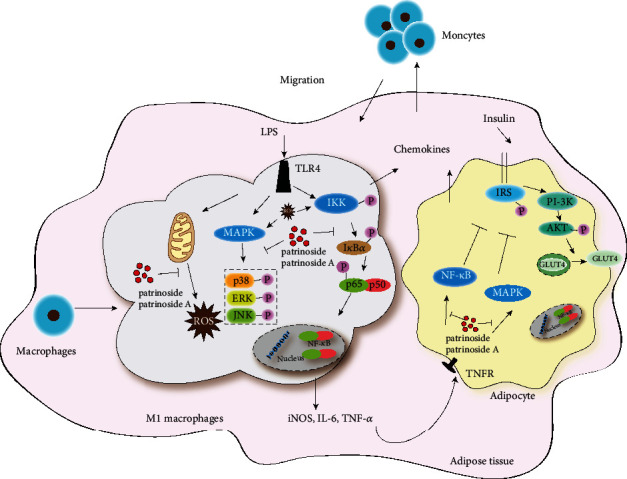
Possible signal transduction pathways of patrinoside and patrinoside A on improving IR.

Above all, these results suggested that patrinoside and patrinoside A could improve IR through inhibiting NF-*κ*B and MAPK signaling pathways, reducing the secretion and transcription of inflammatory cytokines and chemokines, inhibiting macrophage infiltration into adipose tissue, and reducing ROS level (Figure 11).

## 5. Conclusion

Patrinoside and patrinoside A could inhibit the activation of NF-*κ*B and MAPK pathways in macrophage and IR adipocyte inflammation, reduce the transcription and secretion of inflammatory factors such as IL-6 and TNF-*α*, and thus inhibit the infiltration of macrophages into adipocytes, thereby reducing adipocyte inflammation and ultimately improving IR. In addition, patrinoside and patrinoside A also down-regulated the increase of ROS. These results suggested that patrinoside and patrinoside A could improve IR through multi pathways.

## Data Availability

The data used to support the findings of this study are included within the article.
